# Left-sided heart disease and risk of death in patients with end-stage kidney disease receiving haemodialysis: an observational study

**DOI:** 10.1186/s12882-020-02074-3

**Published:** 2020-09-25

**Authors:** Anna Axelsson Raja, Peder E. Warming, Ture L. Nielsen, Louis L. Plesner, Mads Ersbøll, Morten Dalsgaard, Morten Schou, Casper Rydahl, Lisbet Brandi, Kasper Iversen

**Affiliations:** 1 Department of Cardiology, Copenhagen University Hospital Herlev, Borgmester Ib Juuls Vej 1, 2730 Herlev, Denmark; 2 Department of Cardiology, Copenhagen University Hospital Rigshopitalet, Blegdamsvej 9, 2100 København Ø, Copenhagen, Denmark; 3 Department of Cardiology, Endocrinology and Nephrology, Copenhagen University Hospital Nordsjaellands Hospital, Dyrehavevej 29, 3400 Hillerød, Denmark; 4 Department of Nephrology, Copenhagen University Hospital Herlev, Borgmester Ib Juuls Vej 1, 2730 Herlev, Denmark

**Keywords:** Cardiovascular, End-stage renal failure, Dialysis, Echocardiography, Left ventricular systolic dysfunction, Heart failure, Valve disease, Outcome, Mortality, Survival

## Abstract

**Background:**

Cardiovascular disease is the most common cause of death in patients with end-stage kidney disease on haemodialysis. The potential clinical consequence of systematic echocardiographic assessment is however not clear. In an unselected, contemporary population of patients on maintenance haemodialysis we aimed to assess: the prevalence of structural and functional heart disease, the potential therapeutic consequences of echocardiographic screening and whether left-sided heart disease is associated with prognosis.

**Methods:**

Adult chronic haemodialysis patients in two large dialysis centres had transthoracic echocardiography performed prior to dialysis and were followed prospectively. Significant left-sided heart disease was defined as moderate or severe left-sided valve disease or left ventricular ejection fraction (LVEF) ≤40%.

**Results:**

Among the 247 included patients (mean 66 years of age [95%CI 64–67], 68% male), 54 (22%) had significant left-sided heart disease. An LVEF ≤40% was observed in 31 patients (13%) and severe or moderate valve disease in 27 (11%) patients. The findings were not previously recognized in more than half of the patients (56%) prior to the study. Diagnosis had a potential impact on management in 31 (13%) patients including for 18 (7%) who would benefit from initiation of evidence-based heart failure therapy. After 2.8 years of follow-up, all-cause mortality among patients with and without left-sided heart disease was 52 and 32% respectively (hazard ratio [HR] 1.95 (95%CI 1.25–3.06). A multivariable adjusted Cox proportional hazard analysis showed that left-sided heart disease was an independent predictor of mortality with a HR of 1.60 (95%CI 1.01–2.55) along with age (HR per year 1.05 [95%CI 1.03–1.07]).

**Conclusion:**

Left ventricular systolic dysfunction and moderate to severe valve disease are common and often unrecognized in patients with end-stage kidney failure on haemodialysis and are associated with a higher risk of death. For more than 10% of the included patients, systematic echocardiographic assessment had a potential clinical consequence.

## Background

Cardiovascular disease (CVD) is the most common cause of death in patients with end-stage kidney disease (ESKD), accounting for more than half of deaths with a known cause [1–5]. In addition, undiagnosed CVD has been suggested as the underlying cause of unexplained deaths in ESKD [1]. Heart failure, regardless of its cause being reduced systolic function, valve disease or diastolic dysfunction, is characterized by numerous symptoms: dyspnoea, fatigue and ankle swelling. These are all symptoms that can be difficult to distinguish from periodic fluid retention, and therefore development of structural cardiac abnormalities may go unacknowledged in patients with ESKD. Despite the high prevalence of CVD in patients on maintenance dialysis and despite that there is a potential to optimize cardiovascular therapy, there is limited evidence for systematic echocardiographic assessment and to whether this may improve prognosis [6–10]. In an unselected, contemporary population of patients on maintenance haemodialysis we aimed to assess: the prevalence of structural and functional heart disease, the potential therapeutic consequences of echocardiographic screening and whether left-sided heart disease is associated with prognosis.

## Methods

### Study population and design

The study is a cross-sectional, observational study of patients in the two largest dialysis centres (Herlev hospital and Nordsjaellands Hospital) in the capital region of Denmark (1.8 million inhabitants). The Danish healthcare system is publicly funded and provides universal healthcare free of charge, including dialysis service to all citizens. Nearly all Danish hospitals (99% of hospital beds) are public. All patients ≥18 years of age who underwent maintenance haemodialysis from January through April 2014 were eligible and asked to participate. Demographics and medical history were obtained through interviews and supplemented by reviews of medical records including echocardiographic reports (OPUS, version 2.5.0.0,©2010 Computer Sciences Corporation (CSC)).

### Dialysis treatment

Patients followed their routine dialysis treatment and the study made no intervention in treatment. Patients were dialysed on Gambro Artis™ (Gambro AB, Sweden) machines with large, synthetic high flux filters > 1.6 m2. The filters were either Polyamix® (210H or 170H Gambro Polyflux filters) or Polysulfone (Fresenius FX 100, FX 80, or FX 50 filters, Fresenius Medical Care, Germany). The aim of haemodialysis treatment adequacy was to maintain a Kt/V > 1,3/dialysis session. Both haemodialysis and haemodiafiltration were used in individual patients.

### Transthoracic echocardiography

Transthoracic echocardiography (TTE) was performed prior to dialysis (maximum interval 30 min from TTE to initiation of dialysis) using a Vivid S6 ultrasound machine (GE Vingmed Ultrasound A/S, Horten, Norway) by one of three sonographers. M-mode, 2D and Doppler images were performed and analysed according to guidelines from the European Society of Cardiology and American Society of Echocardiography [11, 12]. Primary analyses were done offline post examination by the same sonographer who performed the echocardiography and were reviewed by a cardiologist. Images were analysed using EchoPac software (GE Vingmed Ultrasound A/S, Horten, Norway). Left ventricular (LV) mass was estimated with the Devereux formula. LV volumes, ejection fraction (LVEF) and left atrial (LA) volume ere measured using the biplane method of discs summation (Simpson’s). Both LVEF< 50% and LVEF≤40% were registered because LVEF< 50% was used as definition of reduced ejection fraction/heart failure in medical records, while evidence of heart failure treatment is based on LVEF≤40%. Right ventricular function was measured using the tricuspid annular plane systolic excursion (TAPSE). Images were assessed quantitatively for aortic stenosis and mitral regurgitation and qualitatively for aortic regurgitation and tricuspid regurgitation. Aortic stenosis was evaluated by aortic valve jet maximum velocity and mean gradient using continuous wave Doppler. The aortic valve area was estimated by the continuity equation. Aortic stenosis was defined as mild if max aortic jet velocity was 2.6–2.9 m/s with a mean gradient < 20 mmHg and an indexed aortic valve area of > 0.85 cm^2^/m^2^, as moderate if the indexed aortic valve area was < 0.85 cm^2^/m^2^, the mean gradient 20–40 mmHg or maximum velocity was 3.0–4.0 m/s and as severe if indexed aortic valve area was < 0.60 cm^2^/m^2^, the mean gradient > 40 mmHg or the max velocity > 4.0 m/s. Aortic and mitral regurgitation was documented if considered more than trace regurgitation. Mitral regurgitation was (if suspected more than mild in qualitative assessment) quantified using the PISA-method. Regurgitation was defined as mild if effective regurgitant orifice was < 0.2 cm^2^ or regurgitation volume < 30 mL, as moderate if effective regurgitant orifice was 0.2–0.4 cm^2^ or regurgitation volume 30–60 mL and as severe if effective regurgitant orifice was ≥0.4 cm^2^ or regurgitation volume ≥ 60 mL.

### Exposure, follow-up and outcome

Significant structural or functional left-sided heart disease was defined as moderate or severe aortic or mitral valve disease or LVEF ≤40%. Patients with significant structural or functional left-sided heart disease were compared to those without. Information on vital status and date of death was found through review of medical records. The study population was followed from time of echocardiography until death, emigration, or end of study period, whatever came first.

### Statistics

We included data from all patients with acceptable image quality in the analyses. Normally distributed data are presented as mean (SD) and non-normally distributed data as median (interquartile range). Differences between two groups for continuous variables were compared using the independent Student’s t-test or the Mann-Whitney U test (Wilcoxon rank-sum) depending on distribution of data. Differences between several subgroups for continuous variables were analysed with ANOVA and further explored with the unpaired Student’s t-test if any differences were found. Categorical variables were compared using the Chi squared test. Survival for exposed and non-exposed is shown by Kaplan–Meier curves and difference between groups were assessed using log-rank test. A cox proportional hazard analysis adjusted for age, sex, ischemic heart disease, diabetes, hypertension and dialysis vintage was used to assess hazard ratios with 95% confidence intervals (CI) for exposed compared with non-exposed. A 2-sided *p*-value of < 0.05 was considered statistically significant. The statistical analyses were performed using SAS version 9.3 (Cary, NC, USA).

## Results

### Population characteristics and history of structural heart disease

Informed consent was obtained from 252 of 372 adult patients who received maintenance haemodialysis treatment at the two participating centres. Five patients had to be excluded due to inadequate image quality. In total, 247 echocardiograms were analysed. Patients were 66 (IQR 64–67) years of age and predominantly male (68%). Characteristics of included patients are presented in Table 1. Demographic characteristics were available for all patients. Diabetic nephropathy was more frequent among the included patients (21% vs. 32%, *p* = 0.03), otherwise there were no statistically significant differences in demographic or haemodynamic parameters between included and non-included patients. According to medical records, 38 (15%) patients had a previous history of heart failure and 26 (11%) of valve disease.

**Table 1:** Patient characteristics (*n* = 247)

Age (years), mean (95%CI)	65.6 (63.8–67.4)
Female, n (%)	78 (32)
Body mass index (kg/m^2^) mean (95%CI)	25.5 (24.8–26.2)
History of smoking, n (%)	152 (62)
Vital signs mean (95%CI)	
Systolic BP (mmHg)	144 (141–147)
Diastolic BP (mmHg)	77 (75–79)
Heart rate (BPM)	71 (70–73)
Dialysis, mean (95%CI)	
Dialysis vintage (years)	3.8 (3.3–4.2)
Residual diuresis (mL)	623 (529–717)
Fluid filtration (L)	2.0 (1.9–2.1)
Weekly haemodialysis treatments	3.1 (3.0–3.1) range 2–7.
Cause of kidney failure, n (%)	
Diabetes	49 (20)
Hypertension	50 (20)
Polycystic kidney disease	24 (10)
Glomerulonephritis	27 (11)
Other	97 (39)
Medical history, n (%)	
Diabetes	74 (30)
Hypertension	148 (60)
Ischemic heart disease	65 (26)
Previous stroke	48 (19)
Chronic obstructive pulmonary disease	21 (9)
Heart failure	38 (15)
Valve disease	26 (11)
Atrial fibrillation	52 (21)
Pacemaker	3 (1)
Implantable converter defibrillator	3 (1)
Previous kidney transplantation	5 (2)
Heart transplantation	1 (0.4)
Medical treatment, n (%)	
Diuretics	118 (48)
Beta-blockers	138 (56)
ACE-inhibitor or ARB	54 (22)

All continuous variables are presented as mean and 95% confidence interval (CI), categorical data as number and percentage (%). ACE denotes angiotensin converting enzyme; ARB denotes angiotensin receptor blocker; *BP* pre-dialysis blood pressure, *LVEF* left ventricular ejection fraction

### Echocardiographic findings

#### Ventricular systolic function

Echocardiographic findings are presented in Table 2 as mean values and as percentage of patients with abnormal value. LV systolic function was reduced with an LVEF< 50% in 79 (34%) patients of whom 31 (13%) had an LVEF≤40%. Of the patients with LVEF≤40%, indicating a beneficial effect of heart failure therapy, 19 (61%) did not have a previous history of heart failure. In total, 9% (19/209) of patients with a presumed normal systolic function pre-screening were thus diagnosed through participation in the study. Right ventricular systolic dysfunction, defined as TAPSE < 17 mm was seen in 50 (20%) patients.

**Table 2:** Echocardiographic findings

Parameter	All patients (*n* = 247)	Women (*n* = 78)	Abnormal, n (%)	Reference, women	Men (*n* = 169)	Abnormal, n (%)	Reference, men
Chamber sizes							
LV mass index (g/m^2^)	110 (107–114)	107 (100–113)	45 (58)	43–96	112 (108–116)	70 (42)	49–115
LV diameter index (cm/m^2^)	2.7 (2.6–2.7)	2.8 (2.7–2.9)	16 (21)	2.3–3.1	2.6 (2.5–2.7)	21 (12)	2.2–3.0
LA volume index (ml/m^2^)	35 (33–37)	35 (32–38)	31 (47)	16–34	35 (33–38)	71 (49)	16–34
RV diameter (cm)	2.8 (2.7–2.8)	2.7 (2.6–2.8)	7 (10)	1.9–3.5	2.8 (2.7–2.9)	19 (13)	1.9–3.5
LV and RV systolic function							
LVEF (%)	53 (51–54)	52 (49–54)	41 (58)	54–74	53 (52–55)	63 (40)	52–72
S’lateral LV (cm/s)	7.7 (7.4–8.0)	7.3 (6.8–7.8)	17 (22)	> 5.6	7.9 (7.5–8.2)	26 (16)	> 5.8
TAPSE (cm)	2.2 (2.1–2.2)	2.2 (2.1–2.3)	13 (17)	≥1.7	2.2 (2.1–2.2)	37 (23)	≥1.7
S′ RV (cm/s)	12.7 (12.3–13.2)	12.4 (11.8–13.1)	15 (20)	≥10	12.9 (12.3–13.5)	32 (21)	≥10
PASP (mmHg) (*n* = 172)	37 (35–39)	36 (32–39)	17 (29)	< 40	37 (35–39)	42 (37)	< 40
TR-gradient (mmHg) (*n* = 185)	30 (28–31)	29 (26–32)	33 (53)	< 25	30 (28–32)	77 (62)	< 25

Mean values for each gender as well as reference values and number of patients with abnormal findings are presented. Values were indexed for body surface area using the Du Bois-formula where appropriate. LA denotes left atrium; *LV* left ventricle, *LVEF* left ventricular ejection fraction, *PASP* pulmonic arterial systolic pressure, *RV* right ventricle, *TAPSE* tricuspid annular plane systolic excursion, *TR* tricuspid regurgitation

#### Valve disease

**Fig. 1:**
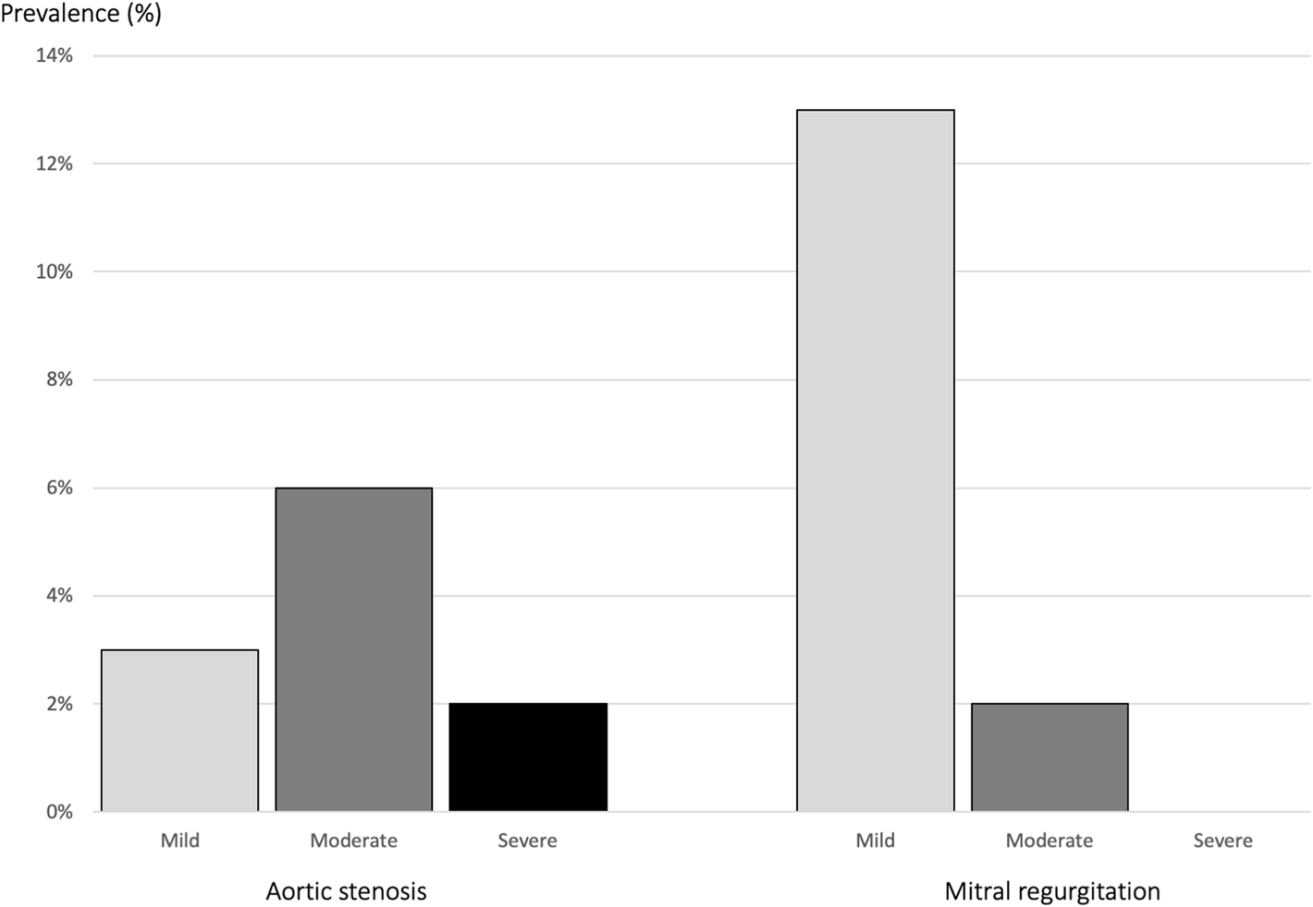
Prevalence and severity of aortic stenosis and mitral regurgitation

Prevalence and severity of aortic stenosis and mitral regurgitation are presented in Fig. 1. Severe aortic stenosis was seen in four (2%) patients, of whom two were previously unrecognized. The two patients with unrecognized severe aortic stenosis were both asymptomatic while the two patients who were previously recognized complained about shortness of breath at moderate exertion. Moderate aortic stenosis was seen in 18 (7%) patients, of whom eight were previously unrecognized. Four of the patients with aortic stenosis had a prosthetic aortic valve without previously recognized prosthetic valve stenosis. Moderate mitral regurgitation was seen in four patients (2%), of whom one was diagnosed prior to inclusion in the study. All four patients with moderate mitral regurgitation had left atrial dilatation, and three had a pulmonary arterial systolic pressure > 50 mmHg. One patient was known to have mitral valve stenosis and was the only patient in the study found with this specific pathology.

### Echocardiographic findings in relation to cause of chronic kidney disease

To explore any differences between different causes of ESKD, we compared patients with an aetiology of hypertension, diabetes, glomerulonephritis and polycystic kidney disease (Table 3). Left atrial volume was significantly larger in patients with hypertensive nephropathy compared to the other groups. We found no difference in the number of patients with LVEF< 50%, LVEF≤40%, or valve disease between groups.

**Table 3:** Echocardiographic findings by cause of kidney disease

Parameter	Diabetic nephropathy (*n* = 49)	Hypertensive nephropathy (*n* = 50)	Polycystic kidney disease (*n* = 24)	Glomerulo-nephritis (*n* = 27)	*P*-value
LV diameter index (cm/m^2^)	2.6 (0.4)	2.6 (0.4)	2.6 (0.4)	2.7 (0.4)	0.7
LV mass index (g/m^2^)	112 (26)	109 (25)	109 (27)	122 (31)	0.2
LA volume index (ml/m^2^)	33 (15)	40 (14)	31 (12)	39 (18)	**0.05**
RV diameter (cm)	2.7 (0.5)	2.9 (0.5)	2.8 (0.5)	2.8 (0.6)	0.5
LVEF (%)	54 (12)	52 (10)	54 (10)	54 (9)	0.9
S’lat LV (cm/s)	7.0 (1.8)	7.4 (2.4)	8.3 (2.5)	8.0 (1.9)	0.08
TAPSE (cm)	2.0 (0.5)	2.2 (0.5)	2.3 (0.6)	2.3 (0.8)	0.1
S′ RV (cm/s)	12.3 (2.9)	12.6 (3.2)	14.3 (4.1)	13.0 (3.6)	0.1
PASP (mmHg)	35 (11)	36 (14)	34 (13)	39 (11)	0.7
TR-gradient (mmHg)	28 (9)	30 (12)	27 (14)	31 (10)	0.7

Variables are presented as mean (SD) and analyzed by ANOVA

LA denotes left atrium; *LV* left ventricle, *LVEF* left ventricular ejection fraction, *PASP* pulmonic arterial systolic pressure, *RV* right ventricle, *TAPSE* tricuspid annular plane systolic excursion, *TR* tricuspid regurgitation

### Clinical consequence of findings at echocardiographic screening

**Fig. 2:**
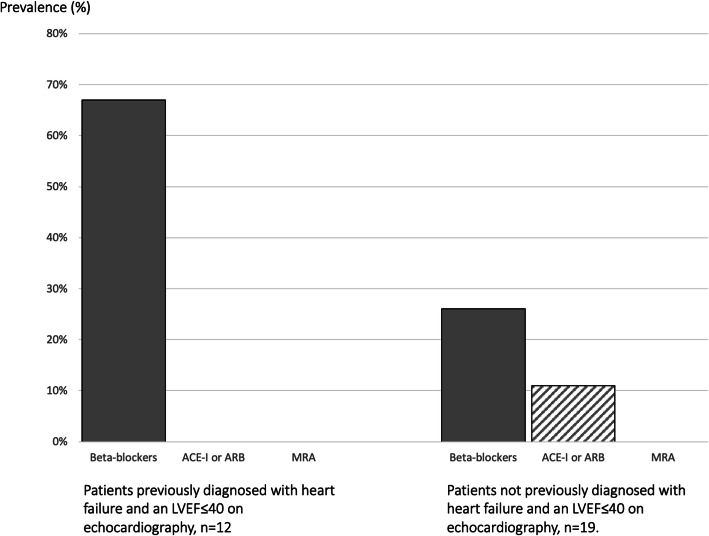
Prevalence of evidence based heart failure treatment in haemodialysis patients with LVEF≤40%. ACE-I denotes angiotensin converting enzyme inhibitor; ARB, angiotensin receptor blocker; MRA mineralocorticoid/aldosterone receptor antagonist; LVEF, left ventricular ejection fraction

Echocardiographic screening could potentially have an impact on management for 31 (13%) of the participating patients. Only one of the 19 patients who were found to have an LVEF≤40% without previously having been diagnosed with a heart failure, received both beta-blockers and angiotensin-converting enzyme (ACE) inhibitors/angiotensin receptor blockers (ARB) (Fig. 2). Thus, 7% of the screened population would potentially benefit from early detection of systolic dysfunction and initiation of evidence- based heart failure therapy. With regards to valve disease, none of the patients who were found with previously unrecognized aortic or mitral stenosis or regurgitation in our study would qualify for valve replacement based on severity and symptoms combined. However, the findings in 13 (5%) patients who were previously unrecognized (two with severe aortic stenosis, eight with moderate aortic stenosis and three with moderate mitral regurgitation) suggest that they should undergo careful clinical and echocardiographic reevaluation at regular or intensified intervals. It was not evident if and why the two patients with symptomatic, severe aortic stenosis had not been evaluated for valve replacement.

### Outcome

**Fig. 3:**
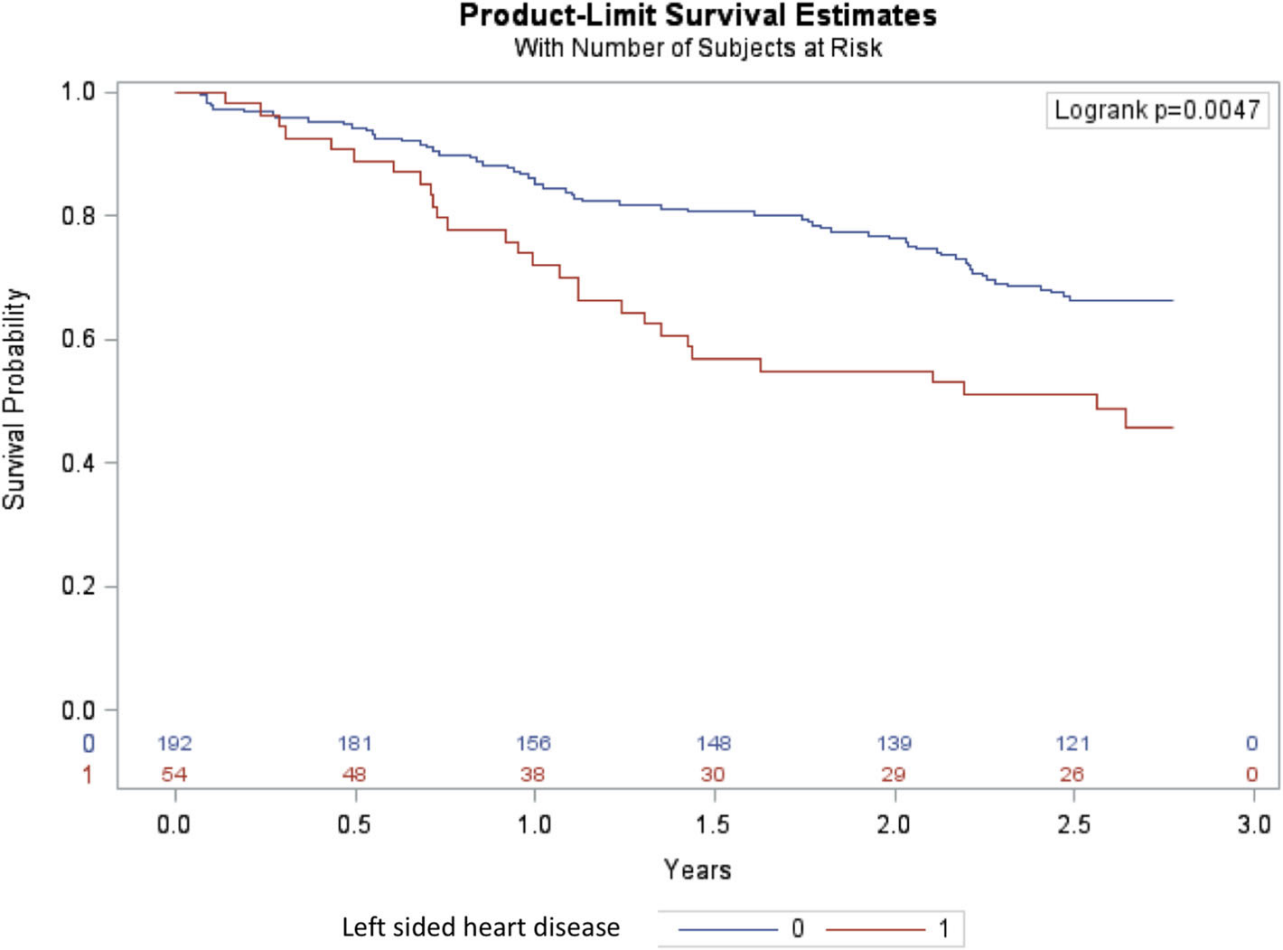
Kaplan-Meier estimate of survival in patients with end stage kidney disease receiving haemodialysis stratified according to presence or absence of left-sided heart disease. Red line illustrates patients with left-sided heart disease and blue line patients without left-sided heart disease

No patients were lost to follow-up. After 2.8 years of follow-up, all-cause mortality among patients with and without left-sided heart disease was 52 and 32% respectively (hazard ratio [HR] 1.95 (95%CI 1.25–3.06) (Fig. 3). A multivariable adjusted Cox proportional hazard analysis including age, sex, ischemic heart disease, diabetes, hypertension and dialysis vintage, showed that structural left-sided heart disease was independently associated with mortality with a HR of 1.60 (95%CI 1.01–2.55) along with age (HR per year 1.05 [95%CI 1.03–1.07]).

## Discussion

We performed systematic echocardiography in a contemporary population of patients on maintenance haemodialysis. The major findings are the following: 1) The prevalence of structural heart disease remains high in the population. One third of patients had LV systolic dysfunction and 10% had moderate to severe valve disease. 2) Left-sided heart disease is often unrecognized: In total, 9% of patients with a presumed normal systolic function pre-screening had an LVEF≤40. To put it differently 61% of the patients with LVEF≤40 were not diagnosed prior to inclusion in the study. 3) In the light of contemporary knowledge, the findings had a potential clinical consequence for 13% of the included patients including 7% who could benefit from detection of systolic dysfunction and initiation of evidence-based heart failure therapy. 4) Left-sided heart disease, defined as ventricular systolic dysfunction and moderate to severe valve disease, was independently associated with a higher risk of death.

Patients with chronic kidney disease (CKD) have a high prevalence of CVD. CKD and CVD share several risk factors, and in addition ESKD exposes the heart to several factors that may accelerate development of CVD [13]. For patients with ESKD specifically the K/DOQI Cardiovascular Disease in Dialysis Patients Clinical Practice Guidelines, recommend echocardiography in all incident patients on dialysis and every three years or when considered for kidney transplantation, but the evidence behind the recommendations remains sparse [14].

The proportion of patients with systolic dysfunction was 34% for LVEF< 50 and 13% for LVEF≤40%, the criteria used in most heart failure trials. In comparison, Yamada et al. found a prevalence of LVEF< 50% in only 13% at initiation of dialysis (*n* = 1254) [9]. In other contemporary studies of patients on already established renal replacement therapy the proportion of patients with systolic dysfunction is similar to the prevalence found in our study. The CRIC study, demonstrated a significant decline in LVEF with progression of kidney disease and found an LVEF< 50% in 48% of 190 patients one year after initiation of dialysis [15]. Assa et al., although excluding patients with severe functional limitation (NYHA IV), found LVEF< 50% in 35% of 105 haemodialysis patients [6]. In a retrospective analysis of 250 patients starting haemo- or peritoneal dialysis, Derthoo et al. described LVEF< 45% in 18% of patients [16].

Several previous studies have found that heart failure or reduced LVEF in patients with ESKD are associated with a poor prognosis with mortality rates comparable to those found in our population [9, 16, 17]. Two-year survival was reduced from 80 to 33% in patients with heart failure and from 73% in patients with LVEF> 45 compared to 55% in those with LVEF ≤45% [16, 17]. The mortality rates in our study after 2.8 years of follow-up were comparable to those studies with all-cause mortality of 52% for patients with significant left-sided heart disease and 32% for patients without.

An important finding of the study was that systematic echocardiography had a potential clinical consequence for 13% of the included patients including 7% who could potentially benefit from detection of systolic dysfunction and initiation of evidence-based heart failure therapy. The finding supports the recommendations by K/DOQI about systematic echocardiography but we cannot conclude from our data that all patients should have an echocardiography performed, because it remains to be elucidated whether taking action on the findings from systematic echocardiography can change prognosis.

Beta-blockers, ACE inhibitors, ARB and mineralocorticoid/aldosterone receptor antagonists (MRAs) convincingly reduce mortality and morbidity in heart failure patients with reduced systolic function in the general population [18]. Heart failure therapy is not based on as strong evidence in patients with ESKD since patients on dialysis are regularly excluded from large clinical trials, but existing data indicate that failure therapy is beneficial and safe [19–29]. At this time, guidelines find no justification for withholding heart failure treatment from patients based merely on kidney function, although the dosing regimen may need individual adjustments depending on side effects including hypotension during dialysis and hyperkalaemia [14, 30]. In spite of these recommendations, not all dialysis patients with heart failure receive adequate heart failure treatment [16, 31]. In our study 67% of patients with previously recognized symptomatic heart failure and an LVEF≤40% received beta-blockers but none were on ACE-I/ARB.

Patients with valve disease should be re-evaluated on a regular basis to recognize progression and initiate timely treatment prior to death or irreversible damage to ventricular function [32]. In patients with ESKD, in addition to an increased mortality, valve disease may impair the ability to deliver adequate dialysis, resulting in suboptimal treatment of both volume overload and toxin removal, potentially contributing further to CVD [14]. Left-sided valve disease was prevalent in our study, although none of the previously undiagnosed patients would have qualified for valve replacement at the time of echocardiography. The prevalence of aortic stenosis (11%) in our study was similar to the prevalence in patients with severe CKD reported by Samad et al. from an extensive echocardiographic database where aortic stenosis was almost three times as prevalent in patients with severe CKD compared to patients with normal kidney function [33]. The prognosis of patients with severe aortic stenosis is poor as soon as symptoms occur, with a 5-year survival of only 15–20% [32]. Consequently, aortic valve replacement is strongly recommended in all patients who are considered to have a life expectancy of > 1 year and a considerable quality-of-life benefit from the procedure, taking their comorbidities into account [34]. Accurate survival estimates are not available for the population of patients with ESKD and concomitant symptomatic left-sided valve disease. Samad et al. found increasingly worse outcomes with increasing severity of valve disease with a five-year survival estimate for patients with CKD and severe aortic stenosis of 42% compared to 67% in patients without CKD. On the other hand, mortality associated to aortic valve replacement is higher in dialysis patients compared to the general population [14, 35]. Treatment of high-risk patients has been revolutionized since the introduction of transcatheter aortic valve replacement (TAVR). As in the large heart failure trials, patients on dialysis were not represented in the large TAVR trials. In a retrospective analysis, the 3-year survival post-TAVR was 30% in patients (*n* = 74) with stage 5 CKD (on dialysis or pre-dialytic) [36]. Based on contemporary knowledge regarding the natural history and outcomes after valve replacement, recommendations for treatment of valve disease in patients with CKD follow those for the general population [14].

The timing of echocardiography relative to dialysis is important since volume overload influences echocardiographic parameters. We performed echocardiography prior to dialysis, in patients that received an average of 3.1 weekly treatments. Present guidelines do not specify the optimal timing of echocardiographic. We have previously shown that echocardiographic evaluation of diastolic function in patients with ESKD is critically dependent on timing relative to dialysis with an improvement of diastolic function after unloading with haemodialysis [37]. However, we did not find a significant change of left ventricular systolic function after haemodialysis. Based on our data, we cannot rule out the possibility that the severity of valve disease was overestimated due to volume overload.

The study has some other strengths and limitations that must be acknowledged: patients were neither selected based on a clinical indication for echocardiography were they previously diagnosed CVD. Furthermore, all patients were assessed with the same standardized protocol by three sonographers within 30 min prior to dialysis. We found no major differences between included and not included patients. Information on the reason for inadequate medication in patients with known heart failure and candidacy for kidney transplantation were not available, which is a potential limitation for the interpretation of the clinical consequences.

## Conclusion

In conclusion, left ventricular systolic dysfunction and moderate to severe valve disease are common and often unrecognized in patients with end-stage kidney failure on haemodialysis and are associated with a higher risk of death. For more than 10% of included patients, systematic echocardiographic assessment had a potential clinical consequence. It remains to be elucidated whether taking action on the findings from systematic echocardiography can change prognosis in these patients.

## Data Availability

The datasets analysed during the current study are not publicly available since individual privacy could be compromised but are available from the corresponding author on reasonable request.

## Abbreviations

ACE: Angiotensin-converting enzyme
ARB: Angiotensin receptor blockers
BP: Blood pressure
CI: Confidence intervals
CKD: Chronic kidney disease
CVD: Cardiovascular disease
ESKD: End-stage kidney disease
HR: Hazard ratio
IQR: Interquartile range
LA: Left atrium
LV: Left ventricle
LVEF: Left ventricular ejection fraction
MRA: Mineralocorticoid/aldosterone receptor antagonist
PASP: Pulmonic arterial systolic pressure
RV: Right ventricle
SD: Standard deviation
TAPSE: Tricuspid annular plane systolic excursion
TAVR: Transcatheter aortic valve replacement
TR: Tricuspid regurgitation
TTE: Transthoracic echocardiography
